# Review of Structural Features and Binding Capacity of Polyphenols to Gluten Proteins and Peptides In Vitro: Relevance to Celiac Disease

**DOI:** 10.3390/antiox9060463

**Published:** 2020-05-29

**Authors:** Miguel Ribeiro, Telma de Sousa, Patrícia Poeta, Ana Sofia Bagulho, Gilberto Igrejas

**Affiliations:** 1 Department of Genetics and Biotechnology, University of Trás-os-Montes and Alto Douro, 5000-801 Vila Real, Portugal; jmribeiro@utad.pt (M.R.); telmaslsousa@hotmail.com (T.d.S.); 2 Functional Genomics and Proteomics Unity, University of Trás-os-Montes and Alto Douro, 5000-801 Vila Real, Portugal; 3 LAQV-REQUIMTE, Faculty of Science and Technology, University Nova of Lisbon, Lisbon, 2829-546 Caparica, Portugal; ppoeta@utad.pt; 4 Microbiology and Antibiotic Resistance Team (MicroART), Department of Veterinary Sciences, University of Trás-os-Montes and Alto Douro (UTAD), 5000-801 Vila Real, Portugal; 5 National Institute for Agrarian and Veterinarian Research (INIAV), Estrada Gil Vaz, Ap. 6, 7350-901 Elvas, Portugal; ana.bagulho@iniav.pt

**Keywords:** polyphenols, gluten, celiac disease, adjuvant therapy

## Abstract

Polyphenols have been extensively studied due to their beneficial effects on human health, particularly for the prevention and treatment of diseases related to oxidative stress. Nevertheless, they are also known to have an anti-nutritional effect in relation to protein metabolism. This effect is a consequence of its binding to digestive enzymes and/or protein substrates. Dietary gluten is the main trigger of celiac disease, a common immune-based disease of the small intestine and for which the only treatment available is the adherence to a gluten-free diet. Recent studies have addressed the use of dietary polyphenols to interact with gluten proteins and avoid its downstream deleterious effects, taking the advantage of the anti-nutritive nature of polyphenols by protein sequestering. Flavonoids, coumarins and tannins have shown the ability to form insoluble complexes with gluten proteins. One of the most promising molecules has been epigallocatechin-3-gallate, which through its binding to gliadins, was able to reduce gliadins digestibility and its ability to stimulate monolayer permeability and transepithelial transport of immunodominant peptides in cell models. This review focuses on the structural features and binding capacity of polyphenols to gluten proteins and peptides, and the prospects of developing an adjuvant therapy in celiac disease.

## 1. Introduction

Celiac disease is an autoimmune disease triggered by the ingestion of wheat gluten and similar proteins found in rye and barley in genetically predisposed individuals [1]. Gluten, the major protein component of wheat endosperm (accounting for about 85% of it) is composed of two types of proteins: gliadins and glutenins. The molecular weight of gliadins ranges from 28 to 55 kDa and they are present essentially as monomers. The glutenins are the polymeric ones, i.e., they can establish both intra and intermolecular disulfide bonds with a molecular weight that can surpasses 34,000 kDa (glutenin macropolymer) [2,3]. The proline-rich nature of gluten proteins is decisive in the survival of some immunogenic peptides to brush-border and luminal protease processing, which ultimately will trigger and maintain the enteropathy in celiac disease. They can directly affect the structure and function of intestinal cells by different molecular mechanisms, such as intracellular tight junction dysfunction, rearrangement of actin cytoskeleton, modulation of gene expression, altered cell differentiation and apoptosis [4].

Contrary to what was initially thought, today it is known that glutenin-derived peptides can also be immunotoxic to celiac disease patients [5]. Nevertheless, gliadins continue to be regarded as the most important proteins for celiac disease as they present the highest immunostimulatory potential [6]. Several studies have shown an unusually long and potent T-cell proliferation agent, the 33-mer peptide from α-gliadins. The proteolytic resistance of this peptide, as well as the fact that it harbors three important T-cell-restricted epitopes (DQ2.5-glia-α1a, DQ2.5-glia-α1b and DQ2.5-glia-α2), make him one of the most important peptides in the pathogenesis of celiac disease [7].

The treatment of celiac disease includes strict adherence to a lifelong gluten-free diet. This diet is effective in most patients, but recurrent or persistent symptoms are common in clinical practice [8]. On the other hand, some nutritional deficiencies have been associated with gluten-free diet, namely iron, vitamin B12, vitamin D, calcium, folic acid and mineral deficiencies [9]. In addition to the nutritional component, celiac patients also present some social vulnerability which often results in the incompliance of gluten-free diet [10]. In this context, alternative approaches need to be addressed to overcome the biopsychosocial burden in celiac disease [11]. Some promising approaches took the advantage of knowing the primary trigger of celiac disease, the gluten, and tried to detoxify it: by means of genetic modification as the genetically modified low-gliadin wheat lines, using gluten specific proteases to deliver a complete digestion of immunogenic peptides, using microbial transglutaminase to hamper the deamidation process performed by tissue transglutaminase (TG2) [5] and even through the sequestration of gliadin proteins or peptides by a novel gluten molecular re-organization [12] or after ingestion by the use of synthetic polymeric binders in order to decrease gliadin digestibility [13]. Despite the promising results, including clinical trials, none of these alternative therapies is being used in celiac disease [5]. This reflects the need for new studies on this issue, so that the adoption of some of the most effective alternatives to the gluten-free diet is a reality in the shortest possible time.

In what concerns the use of polyphenols as potential protective agents within the context of celiac disease, they can be used as natural gluten protein sequestrants in order to avoid or to modulate its immunogenic potential and the panoply of its downstream deleterious effects. These interactions depend on several factors such as the size, type and structure of the proteins and polyphenols, and are also influenced by solution parameters (pH, ionic strength and temperature) [14]. Polyphenols are found throughout the plant kingdom; nevertheless, depending on the phylum under consideration, the type of polyphenols varies considerably. In general, they can be divided into different subgroups by the number of phenolic rings and the structural elements that connect them in phenolic acids, flavonoids, tannins, coumarins, lignans, quinones, stilbenes and curcuminoids [15,16,17]. The growing interest and number of research studies on polyphenols is largely due to the properties and effects related to the human health and well-being. Its broad range of biological activities includes anti-inflammatory [18], anticarcinogenic [19], antioxidant [20], antiallergenic [21], cardiovascular protective [22] and antimicrobial effects [23], among others. Generally regarded as an adverse effect, polyphenols can also present antinutritive properties due to their ability to reduce the digestibility of ingested food proteins, to complex and inhibit digestive enzymes [24]. Although this ability to bind proteins is common to most polyphenols, low molecular weight phenols are generally unable to precipitate protein. One of the most effective protein precipitators are highly polymerized tannins, which through hydrogen bonds and hydrophobic interactions form tannin-protein complexes. They have been traditionally considered as antinutrients because their presence is usually accompanied by a reduced digestibility of protein and a subsequent increase in fecal nitrogen [25].

Nevertheless, in celiac disease this can be a very useful feature. This interaction of gluten proteins and some peptides with a pivotal role in the disease pathogenesis with some polyphenols is being recently and continuously studied. Some important results have already been reported regarding the interaction and potential translational effects for celiac disease [26,27,28,29,30,31,32,33,34,35,36,37,38].

This review aims to bring together all the studies so far on the interaction between polyphenols and gluten proteins, the major outcomes including physicochemical and molecular characterization, and to critically discuss on the usefulness of this approach to the development of novel or adjuvant alternatives to the gluten-free diet in celiac disease.

## 2. Methods

The research documents analyzed in this work were extracted from the Elsevier Scopus and PubMed databases. The search queries (TITLE-ABS-KEY({Celiac disease}), (TITLE-ABS-KEY({Gluten}) and (TITLE-ABS-KEY({Phenols}) were used in February 2020 for collecting academic documents and patents including “celiac disease” and/or “gluten” and/or “phenols” terms in the title, abstract and/or keywords, from the year 2000. Only publications in English were included. The articles from the search were assessed according to document type, language and inclusion in subject category. They were further analyzed and the results were used to write this review.

## 3. Polyphenols-Gluten Interaction: Sequestering of Celiac-Related Immunogenic Peptides

The interaction between gluten proteins and other molecules as an alternative approach or adjuvant therapy in celiac disease is not new and represents a potential approach on the issue [5]. An example is the synthetic polymer poly (hydroxyethylmethacrylate-co-styrenesulfonate) or BL-7010. This polymer binds to α-gliadins with high efficiency at stomach-like and duodenal pH, preventing the formation of gliadin immunogenic peptides by its sequestering [13]. Another example, with the advantage of being used directly in raw foods, ascorbyl palmitate in combination with zinc chloride was proposed as a flour additive to interact with TG2 binding motifs in gluten-derived peptides [39]. More recently, it was showed that the interaction between gluten proteins and the aminopolyssacharide chitosan imposes a different gluten reorganization resulting in novel supramolecular structures. This novel supramolecular architecture decreased gluten digestibility, TG2 activity and the ability of gluten to stimulate a T-Cell-mediated immune response [12,40].

The ability of polyphenols to interact with proteins has been extensively studied. These compounds, ubiquitous in nature, are secondary metabolites of plants implied in defensive mechanisms and thousands of different molecules are known, being the aromatic ring bonded to at least one hydroxyl group the common structural characteristic [14]. The interaction with proteins can occur by non-covalent and/or covalent interactions, which are affected concerning a phenolic compound’s physical-chemical nature, mainly by its size, structural flexibility, quantity of hydroxyl groups and type of side chain [41]. For example, bigger polyphenols and presenting also higher abundance of hydroxyl groups, which provides multiple sites for interaction, are features commonly pointed out that strengthen interaction with proteins [42].

Hydrophobic interactions and hydrogen bonding rule the non-covalent interactions between phenolic compounds and proteins, having also electrostatic interactions, van der Waals forces and π-stacking an important role. Regarding the primary structure of proteins, amino acids with the hydrophobic side chain as alanine, isoleucine, leucine, methionine, phenylalanine, tryptophan, tyrosine and valine are good candidates for interactions mediated by hydrophobic forces, whereas others can interact by hydrogen bonds taking place between the nitrogen or oxygen species of amino acids and the hydroxyl groups of phenolic compounds [14]. In what concerns covalent interactions, polyphenols can undergo a process of enzymatic and non-enzymatic oxidation at alkaline pH, delivering highly reactive quinone radicals which are capable to establish covalent bonds with a variety of nucleophiles, including proteins. Of these, the thiol group of cysteine or ε-amino group of lysine residues are some of the most reactive ones [43].

In celiac disease, the ability of polyphenols to interact with glutamine- and proline-rich proteins of gluten can be beneficial for the patient, since gluten proteins sequestering will potentially limit its harmful effects in the intestinal tract. Either by hampering gluten digestion and the release of immunostimulatory fragments or by preventing gluten recognition by cell binding sites, this interaction has been studied and the most recent works on the subject and the major outcomes are shown in Table 1.

**Figure 1:**
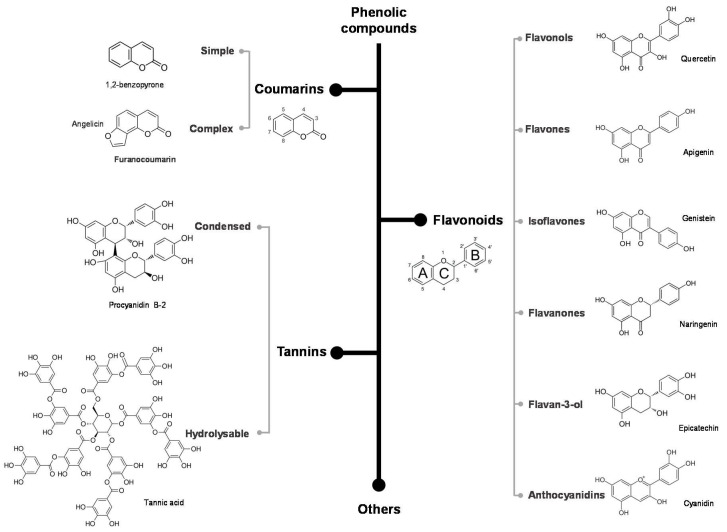
Categories of the principal phenolic compounds involved in the interaction with gluten proteins. Some common molecules are shown.

The principal categories of dietary polyphenols and molecules studied for interaction with gluten, gliadins and/or important immunogenic peptides, namely flavonoids, coumarins and tannins, are schematized in Figure 1 and will be discussed further. Although in the following sections we pointed out important natural sources of different polyphenols that may or may not have the ability to interact with gluten, it should be noted that there is no scientific basis for saying that the simple consumption of foods rich in polyphenols can have a significant impact on the management of celiac disease.

### 3.1. Flavonoids

Flavonoids are the most abundant phenolic compounds in plants [44]. They are low molecular weight, tricyclic compounds with two aromatic rings (ring A and B) linked by a 3-carbon aliphatic chain, which is generally condensed as a pyran (ring C). Within the flavonoids category, these can be ordered by different classes according to the oxidation pattern and central C-ring substitution as follows: flavonols, flavones, isoflavones, flavanones, flavan-3-ols and anthocyanidins (Figure 1) [45]. Briefly, flavonols are the most abundant flavonoids in food, commonly found in onion, apple, lettuce, tomatoes, grapes and other fruits and vegetables, as for example quercetin and kaempferol [46]. They have a hydroxyl group at position 3 of C-ring, which may be also glycosylated [16]. Flavones are less common than other flavonoids and they can be found in red sweet pepper and celery as well as in herbs, grains and cereals. The presence of a double bond between positions 2 and 3 on C-ring and the carbonyl group at position 4 are their common structural features. The luteolin, apigenin and tangeretin are examples of flavones [17]. Isoflavones differ from flavones in the location of B ring, which is attached to the remaining molecule at position 3, showing an estrogen-like structure and can consequently bind to receptors and function as phytoestrogens. They can be found almost exclusively in legumes such as soybeans. Genistin, daidzein and glycitin are some examples of isoflavones [47]. Flavanones are found essentially in citrus fruits. They are distinguished by the presence of a carbonyl group at position 4 of the pyran ring and the absence of the double bond between carbons 2 and 3, i.e., the double bond between positions 2 and 3 is saturated. Naringenin is found in grapefruit, hesperetin in oranges and eriodictyol in lemons [16,46]. Flavan-3-ols, also known as catechins, are distinguished by the presence of a C ring without a carbonyl group at position 4 and absence of the double bond between carbons 2 and 3, presenting instead a hydroxyl group at carbon 3. They are mainly found in drinks, such as green tea and red wine, but also in fruits such as peaches [45]. Anthocyanidins, the aglycone form of anthocyanins, present two double bonds in the heterocyclic ring C, namely between the oxygen and carbon 2 and between carbons 3 and 4. Like flavonols and flavan-3-ols, they have a hydroxyl group at position 3 of C ring. Molecules such as cyanidin, delphinidin, malvidin, pelargonidin and peonidin have been widely studied. They can be found in currants, red grapes, raspberries, strawberries, blueberries and blackberries [48].

Within the category of flavonoids, anthocyanidins, flavan-3-ols and flavonols were the most studied in relation to their binding capacity to gluten proteins [26,27,30,31,32,34,35,36,37,38] (Table 1). The interaction of gluten and its individual fractions with anthocyans was studied mainly by spectroscopic techniques, including ultraviolet–visible (UV-Vis), nuclear magnetic resonance (NMR), infrared (IR) and Raman spectroscopy [31,32,34,35]. All the studies showed a generical molecular interaction between the two moieties and some important features of the interaction were highlighted: (1) in the aglycone forms, no site-preferred interaction was detected, contrarily to their corresponding glycosides, (2) the structure of the anthocyanins had a significant role in their interaction with gluten proteins, and (3) anthocyanins induced conformational changes in gliadins, particularly a significant decrease of the β-turns content. In addition, it should be noted that all the studies were performed using acidic conditions, in a pH range between 2.5 and 4.3. It has been shown that at a lower pH, the interaction between anthocyanins and gluten and its protein groups is favored [31], which seemed to be due to the fact that gluten is more soluble and also adopts a more favorable conformation for the interaction at this pH [31,49,50].

In relation to flavan-3-ols, catechin did not precipitate gluten proteins [30], while epigallocatechin-3-gallate (EGCG) showed a significant capacity to bind to gluten proteins. EGCG interaction was tested with gliadins, gliadin-derived peptides and specific peptides with a pivotal role in the pathogenesis of celiac disease such as 33-mer [7]. Some authors suggested that this interaction is a multi-phase reaction driven by non-specific binding. The initial endothermic phase of the reaction corresponded to hydrophobic interactions followed by a weak exothermic phase driven by hydrogen bonding and finally endothermic reactions culminating in a saturation point. Furthermore, EGCG-33-mer interaction occurred at acidic and basic conditions associated with digestion, a subject that will be discussed further, from the point of view of celiac disease, in Section 4 [37]. Hydrophobic domains were also proposed as primary binding surfaces in peptide-polyphenol complexes, while hydrogen bonding along with van der Waals interactions seemed to stabilize this supramolecular structure [27].

Quercetin was the only tested flavonol as of this review, nevertheless some aspects of the interaction with gliadins have been elucidated and can be important in future studies engaged in developing the strength of flavonoid-gluten proteins interaction. First, pH was considered a determinant aspect because, by influencing the conformation of gliadins, it affects their interaction with flavonol molecules. Hydrophobic interactions seem to rule the association behavior at acidic conditions whereas hydrogen bonds and van der Waals forces take place mainly at higher pH. In relation to the protein secondary structure, quercetin induced a disorder to order transition [38], a behavior already reported for the interaction between EGCG and 33-mer peptide [37]. The tertiary structure was also impacted as gauche-gauche-trans disulfide bond conformations increased at the expenses of the gauche-gauche-gauche conformations [38].

### 3.2. Coumarins

Coumarin (1,2-benzopyrone; Figure 1) consists of fused benzene and pyrone rings [51]. Coumarins and their hydroxylated derivatives are naturally occurring substances in a wide variety of plants, microorganisms and some animal species, both in free and glycosylated forms. They can be divided into simple or complex coumarins, including furano and pyranocoumarins, depending on its chemical structure. Furanocoumarins show a furan ring fused at carbons 6–7 or 7–8, and pyranocoumarins have an additional pyran ring at carbons 7–8. This category of phenolic compounds comprises a vast amount of benzopyrones, being the base compound present in several plants such as green tea, peppermint, blueberry, carrots, among others [52].

A synthetic coumarin, 3-ethoxycarbonylcoumarin (3EcC), was analyzed for the interaction with gliadins using Raman, IR and NMR spectroscopy techniques [35]. As described by other authors for other polyphenols [34,38], the coumarin-gliadin interaction resulted in a more ordered structure of gliadins, with an increase in the α-helix conformation and a decrease in β-sheet and β-turns conformations. The effect was similar to that of a flavonoid (cyanidin), but most important, when the two molecules were used simultaneously, it was found that the presence of both molecules favored this effect [35]. This can open new avenues of research on this topic through the simultaneous use of polyphenols of different categories and nature to enhance their anti-nutritive effect, i.e., protein sequestering in the context of celiac disease.

### 3.3. Tannins

Tannins can be found in blackberries, peaches, cranberries, peas, wine, tea, among other foods [53], being readily distinguishable from other polyphenols by presenting an intermediate to high molecular weight that may be in excess of 30 kDa [54]. They can be divided into two classes, namely hydrolysable and condensed tannins. Hydrolysable tannins are readily hydrolysable molecules and consist of a central monosaccharide molecule, such as glucose, esterified by molecules of gallic acid (gallotannins) or ellagic acid (ellagitannins). Tannic acid is the most common hydrolyzable tannin (Figure 1). Condensed tannins, also known as proanthocyanidins, are polymeric flavans and they often consist in polymers of catechin and epicatechin molecules joined together by carbon-carbon bonds. Unlike hydrolysable tannins, condensed tannins are not readily hydrolysable. Procyanidin B2 is a typic example of a condensed tannin, consisting of two molecules of epicatechin (Figure 1) [54].

Synthetic procyanidins with varying degrees of polymerization (577–1153 Da) and oligomeric procyanidins derived from *Vitis vinifera* grape seeds showed the ability to form stable complexes with different gliadin peptide fractions. These peptide fractions were separated by retention time and were the result of gliadin digestion (pepsin, pancreatin and chymotrypsin), expectably harboring celiac-related immunogenic sequences [29]. At micromolar level, fluorescence assays showed that the size and structure of procyanidins influenced their ability to quench, with a higher Stern–Volmer quenching constant being observed the larger the molecule: oligomeric procyanidins > tetramer TT1 > trimer T1 > B3. On the other hand, at millimolar level, dynamic light scattering showed a main role of the size of peptides, which became a much more decisive driving force when determining the dimension of the resulting aggregates [29]. Some authors reported that the size and structure of both guest and host molecules are important features ruling proanthocyanidins-gluten protein interactions. Proanthocyanidins from sorghum bran and grape seed have bound preferentially to the types of gluten proteins according to their molecular weight and varied as follows: HMW-GS > LMW-GS, ω-gliadins > α-, γ-gliadins. Moreover, besides both sorghum and grape seed proanthocyanidins had shown the ability to precipitate gluten proteins, higher mean degree of polymerization (mDP) sorghum proanthocyanidins (19.5 ± 2.5) presented greater binding affinity for glutenins and gliadins than lower mDP grape seed proanthocyanidins (8.3 ± 0.5) [30].

No binding selectivity for celiac-related toxic sequences was found [28] and a major role of hydrogen bonds in proanthocyanidins-gliadin interactions was suggested [30]. The association characteristics of some flavonoids such as EGCG and some tannins such as procyanidin B3 and C2, with a peptide similar to the immunodominant 33-mer, were also compared, with EGCG being the most reactive polyphenol.

## 4. Potential Translational Effects for Celiac Disease

**Figure 2:**
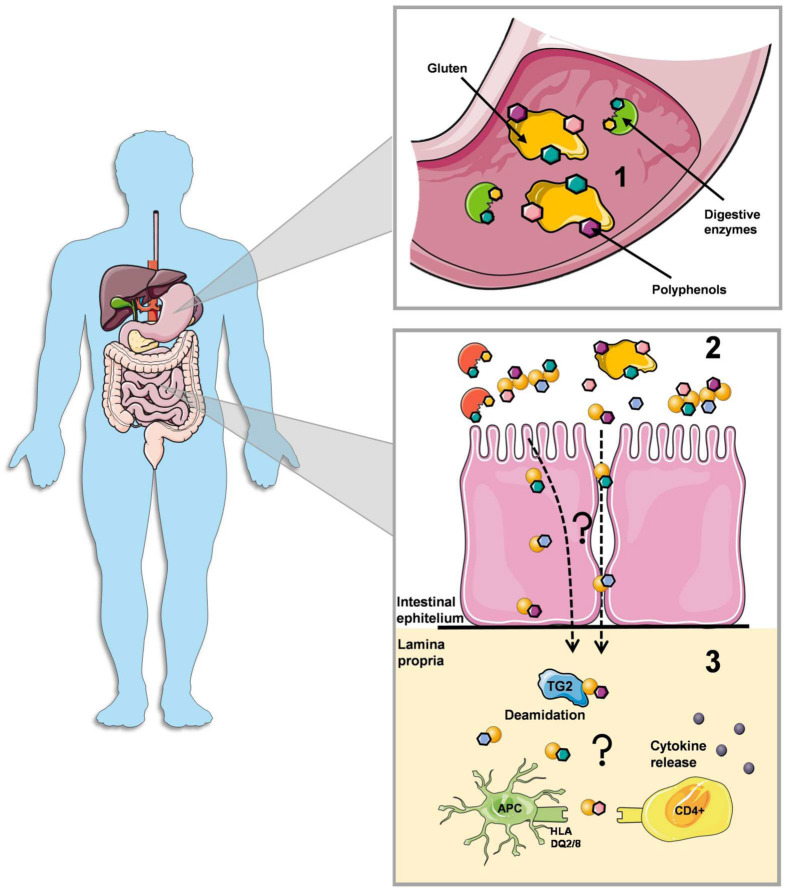
Potential impact of the polyphenols-gluten interaction in celiac disease. (**1**)—Polyphenols can reduce gluten digestibility either by a steric hindrance effect and/or by enzyme activity inhibition. (**2**)—At intestinal epithelium, polyphenols can bind gluten peptides preventing their passage to the lamina propria. The intestinal enzymes can also be inhibited by the interaction with polyphenols preventing further digestion and release of shorter immunogenic peptides. (**3**)—Gluten peptides that reach lamina propria will be deamidated by tissue transglutaminase (TG2) which will enhance the immunostimulatory effect of gluten. Future research should clarify the potential role of polyphenols in the passage of gluten peptides to the lamina propria, in inhibiting TG2 activity and also in the recognition of gluten by antigen presenting cells (APC), which are fundamental processes concerning the adaptive immune response in celiac disease.

Celiac disease involves both adaptive and innate immune systems. In the intestinal epithelium, some gluten peptides can induce tight junction dysfunctions and various cytotoxic effects (apoptosis and altered cell differentiation) [4]. These celiac-related peptides drive the innate immune response, mainly by causing overexpression of interleukin-15 and subsequent activation of intraepithelial lymphocytes that become cytotoxic to enterocytes. When gluten peptides enter lamina propria, they are deamidated by TG2, which converts glutamine residues in glutamic acid residues (Figure 2).

The negative charge imposed by this reaction significantly increases the binding affinity of gluten peptides to HLA-DQ2/8 heterodimeric surface receptors on antigen-presenting cells (APC). In this way, they are presented and properly recognized by CD4+ T cells, resulting in the production of proinflammatory cytokines, the activation of B cells and production of antibodies against gluten and TG2. The eventual consequence is cell damage and villous atrophy mediated by the adaptive immune system processes [1,8]. This knowledge about the pathogenesis of celiac disease allows different therapeutic approaches or approaches aimed at controlling or modulating certain processes (Figure 2). A green tea extract containing mostly EGCG, inhibited pepsin/trypsin-mediated digestion of gliadins in vitro [36]. Moreover, enzyme activity assays showed that green tea extract was also able to inhibit pepsin and trypsin activity in a dose-dependent manner. The authors hypothesized that the inhibition of gliadin digestion can be the result of the physical interaction between green tea extract and gliadins, decreasing gliadins digestibility, the result of direct inhibition of the digestive enzymes or by a combination of these mechanisms [36]. On the other hand, it was demonstrated that EGCG interacts with the proteolytic-resistant and immunodominant peptide 33-mer at a range of pH associated with digestion [37]. Other polyphenols, namely tannins, were also able to interact with some celiac-relevant immunogenic peptides [28], which means that this interaction shows a potential to function as a first defense post-ingestion system, decreasing gluten digestibility and most likely in terms of its recognition with regard to innate immune response.

In normal conditions, the intestinal epithelium is basically impermeable to macromolecules like gliadins but in celiac disease paracellular permeability is augmented and the integrity of the tight junction system is compromised [55]. Many studies demonstrated that some polyphenols, particularly flavonoids as quercetin, naringenin, hesperetin and kaempferol, enhance barrier function in human intestinal cells. These polyphenols seem to modulate the expression and/or localization of intercellular tight junctions’ proteins in the cells [56,57]. Considering the importance of the epithelial barrier of the intestine in the passage of immunogenic peptides for the initiation of adaptive immune responses, an important role of polyphenols in containing the development and maintenance of enteropathy in celiac disease may be suspected (Figure 2). Although more studies are needed, some promising results have been recently reported regarding transepithelial transport of gliadins using in vitro cell models. It was demonstrated that the interaction between EGCG and a synthetic peptide similar to the immunodominant 33-mer (32-mer), was able to reduce 32-mer transepithelial transport in an in vitro model of intestinal epithelial barrier using Caco-2 cells [26]. Accordingly, further studies showed a protective effect of EGCG-rich green tea extract against gliadin-mediated intestinal permeability and inflammation. First, the treatment with green tea extract did not exert any significant effect on cell viability, but inhibited gliadin-mediated permeability on Caco-2 cells as transepithelial electrical resistance (TEER) was preserved. Noteworthy, the secretion of proinflammatory cytokines (IL-6 and IL-8) stimulated by the presence of peptic/tryptic digests of gliadin was also mediated by the green tea extract, a protective effect that was sustained over 24 h [36]. Like EGCG, tannins like procyanidin B3 and the trimer C2 also showed the ability to reduce 32-mer transepithelial transport; trimer C2 treatment reduced about 99% the concentration of 32-mer peptide in the basolateral compartment. Furthermore, similar affinity constants were observed for both tannins, a measure that can correlate with the binding strength. Nevertheless, considering the different reduction values in relation to the apical-to-basolateral transport of the 32-mer as procyanidin B3 showed a reduction of about 51%, it was hypothesized that the occurrence of additional mechanisms beyond physical interaction by polyphenols might modulate the transepithelial transport of celiac-related peptides [27].

In addition to the coating effect, the structural modification of the immunostimulatory peptides, such as 33-mer, for blocking its recognition could also be an important asset of the polyphenols-gluten interaction in the context of celiac disease pathogenesis. In particular, this interaction induced a disorder to order transition, being mostly reported an increase in the content of α-helix structures at the expense of β-turns of gliadins [34,35,38]. The disulfide bond conformation was also modified [35,38], which in combination with the secondary structure modifications, can give immunogenic proteins/peptides a specific conformation allowing or not its proper recognition. At least, these structural characteristics play an important role in the allergenicity of gliadins and by modifying these elements, the allergen’s immunoreactivity could be reduced, which could have other implications for other gluten-related disorders in addition to celiac disease [33]. The potential inhibition of the deamidation reaction of gluten peptides carried out by TG2, either by a steric hindrance effect and/or by enzyme activity inhibition, eventually induced by the interaction with polyphenols, is one aspect that needs to be further elucidated. Other aspects relying on the role of phenolic compounds on the recognition of immunostimulatory peptides by APC and the downstream-related effects also deserve our attention. In what concerns the adaptive response in celiac disease, little is known about the effect of these molecules on the most well-known processes, which should be addressed by future research (Figure 2).

## 5. Final Remarks and Perspectives

Polyphenols, namely flavonoids, coumarins and tannins, showed the ability to interact with gluten proteins and specific celiac-related immunodominant peptides to an extent that could allow them to be used as protective agents within the context of celiac disease, but that could hardly allow gluten to be ingested by patients suffering from celiac disease, i.e., as an alternative to the gluten-exclusion diet. At this standpoint, as a post-ingestion therapy, they may have an important role in any event of accidental gluten ingestion, and could, in fact, through their association with it, in small quantities, to reduce gliadin digestibility and eventually to block immunostimulatory peptide recognition, diminishing its effect on the development of symptoms, as well as its earlier control.

An important part of the studies has been focused on the use of EGCG, which showed a significant gluten protein-binding capacity. It was able to reduce gliadin digestion, either by steric hindrance and/or direct inhibition of the digestive enzymes, to reduce gliadin-stimulated monolayer permeability and the release of proinflammatory cytokines by Caco-2 cells. Further investigations should comprise another type of experimental design, including animal models or intestinal cell lines derived from biopsy specimens of celiac patients, in order to obtain results from experimental models closer to the disease pathogenesis process and create bases to putative clinical trials. Additionally, the interaction with other proteins and other nutrients at the gastrointestinal level must be addressed by future research. On the one hand, competitive studies are necessary to understand the capacity of polyphenols to bind to gluten proteins in complex mixtures and at what dose they would be effective in neutralizing gluten immunogenic properties. On the other hand, specificity for gluten should always be a priority, which can potentially be enhanced by the use of other phenolic compounds not yet studied and with another natural or synthetic molecular structure. Mainly due to the fact that it is a recent line of research, there are still many polyphenols to be tested. Nevertheless, the new approach reviewed in this work has already shown promising results and could be another contribution towards solving the biopsychosocial puzzle that is celiac disease.

## Figures and Tables

**Table 1:** An overview of the major studies on the interaction between gluten proteins and phenolic compounds, and its relevance to celiac disease.

Type	Name	Source	Gluten Target Studied	Techniques	pH of Interaction	Major Outcomes	Potential Translational Effects for Celiac Disease	Ref.
Flavonoids	Delphinidin 3-*O*-glucoside chloride (Myrtillin)	Commercial	Gliadins (extracted with ethanol)	UV–Vis, NMR and IR spectroscopy	2.5	Data seem to indicate a generical molecular interaction between the two moieties. Hydrogen bonding is not excluded as well. In the aglycone forms, Cyanidin and Pelargonidin, no site-preferred interaction was detected, contrarily to their corresponding glycosides, Keracyanin and Callistephin	Not studied	[32]
	Cyanidin-3-*O*-β- rutinoside chloride (Keracyanin)							
	Malvidin-3,5-di-*O*-glucoside chloride (Malvin)							
	Pelargonidin-3-*O*-glucoside chloride (Callistephin)							
	Cyanidin chloride (Cyanidin)							
	Pelargonidin chloride (Pelargonidin)							
Flavonoids	Delphinidin-3-*O*-β-d -glucoside chloride (Dp3glu)	Commercial and extracted from eggplants (delphinidin derivatives)	Wheat gluten, gliadins and glutenins (residue remaining after the separation of the gliadin fraction from vital gluten)	UV–Vis spectroscopy; adsorption kinetics and isotherms	3.0 and 4.3	Both commercial and extracted from plants anthocyanins were adsorbed by gluten and its constituent fractions; results indicate an important role of anthocyanins structure in their adsorption by gluten. Dp3glu is more adsorbed than Cy3rut. pH 3.0 favors anthocyanin adsorption because gluten is more accessible	Not studied	[31]
	Cyanidin-3-*O*-β-d -rutinoside Chloride (Cy3rut)	Commercial and extracted from cherries (cyanidin derivatives)						
Flavonoids	Cyanidin 3-*O*-glucoside chloride (Kuromanin)	Commercial	Gliadins (extracted with ethanol)	Raman spectroscopy	2.5	Anthocyanins induced conformational changes in gliadins. Malvin induced the most significant conformational rearrangements, namely a significant decrease of the β-turns content. Pelargonin showed a similar but less pronounced effect as malvin	Not studied	[34]
	Pelargonidin 3-*O*-glucoside chloride (Callistephin)							
	Malvin 3-*O*-glucoside chloride (Oenin)							
	Cyanidin-3,5-di-*O*-glucoside chloride (Cyanin)							
	Pelargonidin-3,5-di-*O*-glucoside chloride (Pelargonin)							
	Malvidin-3,5-di-*O*-glucoside chloride (Malvin)							
Flavonoids	Cyanidin chloride (Cyanidin)	Commercial	Gliadins (extracted with ethanol)	Raman, IR and NMR spectroscopy	2.5 and 7.0 (only 3EcC)	Cyanidin and 3EcC produced a similar effect on the gliadins structure: increase in the α-helix conformation and a decrease in β-sheet and β-turns conformations. The presence of both molecules favored this effect. Gliadins disulphide bond conformation was altered upon interaction with 3-EcC	Not studied	[35]
Coumarins	3-ethoxycarbonylcoumarin (3EcC)	Synthesized						
Tannins	Procyanidin B3	Synthesized	Peptide fractions with different retention times obtained from gliadin digestion (pepsin, pancreatin and chymotrypsin)	Fluorescence quenching measurement and DLS	Experiments were performed in ultrapure water	A stable complex is formed between procyanidins and gliadin peptides. Fluorescence studies indicated an important role of procyanidins degree of polymerization whereas DLS results suggested that the interaction is mainly dependent on the peptide size	Not studied	[29]
	Procyanidin trimer T1							
	Procyanidin tetramer TT1							
	Oligomeric procyanidins	Extracted from *Vitis vinifera* grape seeds						
Tannins	Procyanidin B3	Synthesized	Peptide fractions with different retention times obtained from gliadin digestion (pepsin, pancreatin and chymotrypsin) and a synthetic 32-mer peptide (33-mer peptide without the initial leucine residue)	ESI-MS	Experiments were performed in ultrapure water with 0.1% of formic acid	There was no binding selectivity of procyanidin B3 towards peptide fragments harboring celiac disease T-cell epitopes	Procyanidin B3 interacted with some celiac-relevant immunogenic peptides	[28]
Several *	Mixture	Extracted from artichoke leaf	Gliadins (extracted with ethanol)	SDS-PAGE, RP-HPLC, dot blot inhibition assay of IgG and IgE binding and basophil activation assay	Experiments were performed in 0.05 mol/L acetic acid	Polyphenol extracts from artichoke leaves, cranberries and apples formed insoluble complexes with gliadins. Only cranberry extract decreased gliadin recognition by IgG and IgE and prevented the degranulation process in mast cells	Not studied	[33]
		Extracted from cranberry						
		Extracted from apple						
		Extracted from green tea leaf						
Tannins	Mixture (Proanthocyanidins)	Extracted from high tannin sorghum bran	Gluten, gliadins (extracted with 1-propanol) and glutenins (extracted with 1-propanol with dithiothreitol) from a weak and strong gluten wheat flours	Nephelometry, RP-HPLC, lab-on-a-chip electrophoresis and surface hydrophobicity studies	5.0	Proanthocyanidins—gluten-protein interactions depended on the protein molecular weight and structure, as well as the proanthocyanidins degree of polymerization, increasing with the increase of these characteristics; both extracts precipitated glutenins more efficiently than gliadins. Catechin did not precipitate gluten proteins. Data suggested a main role of hydrogen bonds in proanthocyanidins-gliadin interactions, whereas interaction with glutenins seemed to involve cooperative hydrogen bonding and hydrophobic interactions	Not studied	[30]
		Commercial (from grape seed)						
Flavonoids	Catechin	Commercial						
Flavonoids	Epigallocatechin-3-gallate (EGCG)	Commercial	Synthetic 32-mer peptide (33-mer peptide without the initial leucine residue)	1D and 2D ^1^H NMR, ITC, molecular dynamics simulations and in vitro transepithelial transport assays using a Caco-2 cell line model	7.4	EGCG showed high reactivity towards the 32-mer peptide. 32-mer peptide behaved as a multivalent receptor to EGCG. pH (due to absence of electrostatic repulsions), hydrophobic interactions and the presence of a galloyl group in EGCG molecules along with the hydroxylation pattern of its aromatic rings were highlighted	In vitro transepithelial transport assays using a Caco-2 cell line model, i.e., to simulate intestinal epithelial barrier, showed that EGCG – 32-mer interaction reduced the amount of 32-mer available in the basolateral compartment	[26]
Several *	Mixture (mostly flavonoids with 51% EGCG)	Green tea extract (commercial)	Gliadin (commercial) and pepsin/trypsin-digested gliadin (PT-gliadin)	In vitro digestion and inhibition of proteases activity, SDS-PAGE, turbidimetry, in vitro intestinal permeability and quantification of inflammatory markers in Caco-2 cells	6.8	Gliadin and PT-gliadin interacted with green tea extract, resulting in the formation of insoluble aggregates. Green tea extract reduced pepsin and trypsin protease activity and gliadin digestion	Green tea extract was able to reduce gliadin digestion and reduced gliadin-stimulated monolayer permeability and the release of proinflammatory cytokines IL-6 and IL-8 by Caco-2 cells	[36]
Flavonoids	EGCG	Commercial	Synthetic 33-mer peptide	DLS, ITC, NMR and CD	6.8; 2.0, 6.8 and 7.5 (DLS and CD)	EGCG interacted with 33-mer in a multi-phase reaction driven by non-specific binding and resulted in the formation of polydisperse complexes; interaction induced changes in peptide structure (disorder to order transition). Initial endothermic phase of the reaction corresponded to hydrophobic interactions followed by a weak exothermic phase driven by hydrogen bonding; further reactions were endothermic and culminated in the reaction reaching a saturation point	EGCG-33-mer interaction occurred at a range of pH associated with digestion (2.0, 6.8 and 7.5)	[37]
Flavonoids	Epigallocatechin (EGC)	Commercial	Synthetic 32-mer peptide (33-mer peptide without the initial leucine residue)	NMR, fluorescence quenching measurement, DLS, molecular dynamics simulations, in vitro transepithelial transport assays using a Caco-2 cell line model and LC-MS	7.4	EGCG was the most reactive polyphenol at physiological pH and temperature. Leucine-, tyrosine- and phenylalanine-containing domains were proposed as primary binding surfaces. Hydrophobic interactions were primarily responsible for the binding process between the 32-mer and polyphenols while hydrogen bonding along with van der Waals interactions seemed to stabilize the complexes	Procyanidin B3 and trimer C2 reduced transepithelial transport (only these polyphenols were tested)	[27]
	EGCG							
	Catechin							
Tannins	Procyanidin B3	Synthesized						
	Procyanidin C2							
Flavonoids	Quercetin	Commercial	Gliadins (commercial)	Fluorescence, UV-visible, FTIR and Raman spectroscopy	2.0 to 9.0	pH influenced gliadin conformation and its interaction with quercetin; hydrophobic interaction was predominant at pH 2.0–4.0, whereas hydrogen bonds and van der Waals forces at pH 5.0–9.0. Data showed an increase of α-helix and β-sheet conformations at the expenses of β-turn and random coil conformations; gauche-gauche-trans disulfide bond conformations increased at the expenses of the gauche-gauche-gauche conformations	Not studied	[38]

* Several, mixture of different types of polyphenols; UV–Vis, ultraviolet–visible; NMR, nuclear magnetic resonance; IR, Infrared; DLS, dynamic light scattering; ESI-MS, electrospray ionization mass spectrometry; SDS-PAGE, sodium dodecyl sulfate-polyacrylamide gel electrophoresis; RP-HPLC, reversed-phase high performance liquid chromatography; ITC, isothermal titration calorimetry; CD, circular dichroism; FTIR, Fourier transform infrared spectroscopy.
